# Unrecognized congenital heart disease in rural school-age children: getting to the root of the problem

**DOI:** 10.1007/s12519-022-00538-0

**Published:** 2022-04-06

**Authors:** Qu-Ming Zhao, Guo-Ying Huang

**Affiliations:** 1 Pediatric Heart Center, Children’s Hospital of Fudan University, National Children’s Medical Center, 399 Wan Yuan Road, Shanghai, 201102 China; 2 Shanghai Key Laboratory of Birth Defects, Shanghai, China

Congenital heart disease (CHD) is an important contributor to morbidity and mortality in children, and the global burden of this illness has raised unique challenges to the healthcare system [1, 2]. China is among the countries with the highest burden of this disorder despite having achieved huge advances in cardiac surgery or interventional therapies in CHD during the past two decades [3]. Interestingly and unexpected is the lower prevalence rate of CHD reported in China. The prevalence of CHD at live birth is estimated to be 9.4 per 1000 worldwide [4]. However, this figure is much lower in China, with only 4.9 per 1000 live births between 2015 and 2019 [5].

Furthermore, contrary to the general understanding [2], rural areas in China have a lower CHD prevalence than urban areas [5]. This counterintuitive discrepancy is likely attributed to unrecognized cases of CHD [3], which is still a major public health burden in many developing countries [6–8]. Even though neonatal CHD screening has been adopted for the universal newborn screening program in China since 2018 [10, 11], a longer period may be required for its benefits to come to fruition in rural areas. Late presentation with severe complications (such as heart failure, endocarditis, or irreversible pulmonary hypertension) due to delayed diagnosis has been known to contribute significantly to adverse outcomes, which may largely explain the high disease burden in these settings. Accurate estimates of unrecognized CHD, thus, could contribute to quantifying unmet health needs for diagnosis and treatment.

School age has been suggested to be a period optimal for CHD screening to detect previously unrecognized CHD in rural areas [6, 12]. However, data on the epidemiological survey of CHD among school-age children are still sparse. We are aware of only five studies that have reported the CHD prevalence among school-age children in the whole population, ranging from 0.5 to 2.1 per 1000 [14–18]. As to unrecognized CHD, the global estimate in a recent systematic review was 1.4 per 1000 [13]. Of note, only nine studies were available for this estimate, with four of them from Thailand. Nonetheless, almost all studies have used a traditional survey strategy, where an echocardiographic examination was performed only in suspected cases after clinical assessment. In China, the largest survey of 540,574 students conducted in urban areas has reported a prevalence of 2.1 per 1000, but with only 2.7% undergoing echocardiography [15]. This would inevitably lead to an underestimation of the true prevalence of CHD. On the other hand, although four studies based on a systematic echocardiographic approach reported a higher prevalence (ranging from 6.6 to 19.6 per 1000) [19–22], these studies were sample surveys with a limited sample size between 357 and 4213.

Although less likely to be practical or affordable under most current health care systems, echocardiographic screening of all children would provide an accurate estimate of the unrecognized CHD. Our recent work provided such an estimate in a selected rural area through echocardiographic screening of 21,861 children aged 5–18 years in one town and four townships in Luchun County (totaling 99.2% of all school-age children) [23]. Among the 285 children with CHD identified in the study, only 33 already had parent-reported CHD; the remaining 252 were unrecognized cases. In other words, these 252 were diagnosed for the first time during our screening. Thus, our data showed a prevalence rate of 11.5 per 1000 children, tenfold the global estimate of unrecognized CHD based on the traditional survey strategy. Moreover, unlike previously thought, more than 70% of unrecognized CHD were moderate cases who are physically appreciable and require treatment or were severe cases who already had heart failure symptoms, including cyanosis, fatigue, decreased activity, and growth retardation.

Ethnic disparities have been reported in CHD, although the cause is unclear. Viewed in this light, estimates from this first-ever, population-based echocardiographic study of predominant Hani school-aged children (88.8%) warrant further investigation by screening different populations or ethnicities. Of note, the prevalence of unrecognized atrial septal defect (ASD) is unexpectedly high in the Hani population. Echocardiography can certainly detect more ASD, which may go unnoticed during physical examinations. Still, we cannot rule out the possibility that ASD as a subtype may be more frequent in this ethnicity. Nevertheless, we expect there will not be much difference in CHD as a whole, considering that ethnic differences in CHD prevalence have been reported to be mainly reflected in specific subtypes. If this was the case, we speculate that the overall rate of unrecognized CHD in other rural areas in China would be higher than that in Luchun County, considering that the rate of 11.5 per 1000 of unrecognized CHD was set in the context of the previous five CHD screening campaigns conducted by other medical terms.

Relative to ethnicity, we suspect that socioeconomic status was likely to play a major role in the epidemiology of unrecognized CHD. Our study showed that high-poverty areas and poor households were both critical risk factors for unrecognized CHD, probably explaining the lower CHD prevalence in rural China found in earlier studies [5]. This further supports the notions that limited access to health care and lack of resources and referral systems likely lead to no chance for prompt diagnosis of CHD [3].

Underestimation of unrecognized CHD exists in deprived areas of many other developing countries where newborn screening needs to be improved in many ways, including perfecting the use of the pulse oximetry with a physical examination that has been successfully introduced in China [24]. In view of this, accurate estimates of CHD prevalence in these areas are crucial for policy-makers to plan health care programs and to improve current services. What deters such efforts is the currently suboptimal surveillance and inadequate awareness of CHD among community doctors [8]. Our study’s high prevalence of unrecognized CHD underscores the importance of developing local training programs and increasing awareness of cardiac examinations into compulsory school health screening to reduce the disease burden. In effect, we found that the CHD birth prevalence was doubled (8.9 per 1000) after optimizing cardiovascular examination training in primary hospitals and intensifying follow-up [9]. The challenge facing the policy-makers is how to solve the problem of the disparity in medical resource distribution to allow more patients to benefit from the advancements in CHD diagnosis and treatment. In addition, universal education of medical science knowledge and increasing the awareness of unrecognized CHD are also important public health priorities that should be put on the agenda for health policymaking.
